# Perspectives of Circadian-Based Music Therapy for the Pathogenesis and Symptomatic Treatment of Neurodegenerative Disorders

**DOI:** 10.3389/fnint.2021.769142

**Published:** 2022-01-26

**Authors:** Arastu Sharma, Eric Moon, Geunhoo Kim, Sung-Ung Kang

**Affiliations:** ^1^Neuroregeneration and Stem Cell Programs, Institute for Cell Engineering, Johns Hopkins University School of Medicine, Baltimore, MD, United States; ^2^Peabody Institute Baltimore, Johns Hopkins University, Baltimore, MD, United States; ^3^Department of Neurology, Johns Hopkins University School of Medicine, Baltimore, MD, United States

**Keywords:** music therapy, circadian rhythm, neurodegeneration, Alzheimer’s disease (AD), Parkinson’s disease (PD)

## Abstract

Music therapy (MT) and other rhythmic-based interventions for the treatment of neurodegeneration (ND) have been successful in improving the quality of life of affected individuals. Music therapy and rhythm-based stimuli affect patients with Alzheimer’s disease (AD) and Parkinson’s disease (PD) respectively not only through cognitive channels and subjective qualifications but also through altered brain structures and neural systems. Often implicated in the pathogenesis and resulting symptoms of these diseases is the role of aberrant circadian rhythmicity (CR), namely disrupted sleep. Recent literature suggests that proper maintenance of this timekeeping framework may be beneficial for patients with neurodegenerative disorders and serve a neuroprotective role. While music therapy can improve the quality of life for neurodegenerative patients, longitudinal studies analyzing sleep patterns of affected individuals and possible mechanisms of intervention remain sparse. Furthermore, the role of music therapy in the context of circadian rhythmicity has not been adequately explored. By analyzing the links between circadian rhythmicity, neurodegeneration, and music therapy, a more comprehensive picture emerges, suggesting that possible uses of non-pharmacological circadian-based music therapy to target mechanisms involved in the pathogenesis of Alzheimer’s disease and Parkinson’s disease may enhance clinical treatment and potentially indicate neuroprotection as a preventative measure.

## Introduction

The fundamental understanding and use of music as a therapeutic intervention has experienced countless advancements in recent years. Some of the most noticeable successes have occurred by ameliorating symptoms of patients afflicted with neurodegenerative disorders, namely AD and PD. Bi-weekly drumming has been shown to aid PD patients (Pantelyat et al., 2016), and familiar music has also been shown to improve symptoms of AD patients (Leggieri et al., 2018). However, rhythmicity is not endemic only to music; circadian systems are the embodiment of living rhythmicity, forging their own biologically relevant pulses to sustain life from the molecular to the systematic level. This rhythmicity is an integral component of physiological systems and serves an ever-increasingly recognized role in ND (Sharma et al., 2020), paving the way for the inclusion of music-based treatments.

## Biological Aspect of CR and ND

The basic molecular component of the mammalian biological clock which gives rise to tau, or the free-running period, exists as a negative feedback loop. BMAL1 and CLOCK, two heterodimerization transcription factors, promote the transcription of Per and Cry (Sharma et al., 2020). The produced PER and CRY proteins heterodimerize and are further phosphorylated by casein kinase 1 (CK1), allowing PER/CRY translocation to the nucleus, where the negative feedback loop is initiated when the PER/CRY heterodimer inhibits CLOCK/BMAL1, abating further transcription of PER and CRY until the cycle resets (Cox and Takahashi, 2019; Philpott et al., 2020). Atypical CR in the form of altered sleep-wake cycles or disrupted metabolic patterns may influence the development of cardiovascular diseases, cancer, and even neurodegenerative pathology (Wilking et al., 2013).

Aberrant CR can be observed in ND specifically through the pathogenesis of AD and PD. AD and PD pathogenesis is believed to encompass severe protein aggregation promoting cytotoxicity, cellular stress, and ultimate destruction of neuronal cells (Sharma et al., 2020). The main culprits in the pathogenesis of AD are Tau protein accumulation and β-amyloid (Aβ) aggregation, resulting in widespread atrophy of brain areas as well as degradation of the SCN (Huseby et al., 2019); PD involves a-synuclein aggregation and results in a more targeted degradation of dopaminergic (DA) neurons (Delenclos et al., 2019). CR, primarily sleep-wake cycles, are highly interrelated with these forms of protein aggregates; disrupted sleep is an indicative comorbidity of neurodegenerative diseases and may contribute to the pathology of ND (Sharma et al., 2020). The prevalence of AD and PD is often observed in older age, and Floyd et al. (2000) indicate the prevalence of sleep decline throughout aging in a meta-analysis focusing on sleep quantity and quality over human lifespans; waking frequency and sleep latency increased, as well as reduced sleep. Surprisingly, melatonin administration has been shown to have neuroprotective effects on DA neurons and defensive qualities against Aβ toxicity (Radogna et al., 2010; Wilking et al., 2013). The effects of sleep-wake changes can be predictive of cognitive decline and decreased quality of life in ND (Roh et al., 2012), but further longitudinal studies are required to analyze preventative pharmacological and non-pharmacological treatment (Naismith et al., 2011; Wilking et al., 2013). Aβ levels, already exhibiting circadian fluctuations, have been implicated bidirectionally in the process of sleep, particularly in early and pre-onset AD (Sharma et al., 2020). Cyclic levels of orexin, a wakefulness-promoting neuropeptide crucial for the balance of the sleep-wake cycle, are inversely proportional to tau aggregation, and high levels of Aβ and tau aggregation disrupt non-REM sleep phases (Liu et al., 2019). Inhibition of orexin led to reduced wakefulness and lower levels of Aβ accumulation (Liu et al., 2019). Conversely, excessive daytime sleepiness can promote Aβ accumulation, and particularly slow-wave sleep (SWS) is responsible for boosting glymphatic flow (GF) in mice by 60%, while GF is an imperative process for the clearance of Aβ in interstitial fluid in the central nervous system (Rasmussen et al., 2018). Proper sleep regulation is crucial for the maintenance of benign Aβ activity, and further longitudinal studies on daytime sleepiness in relation to Aβ accumulation and quality of SWS sleep are required to analyze the effects of sleep aberrancy and Aβ pathology (Winer and Mander, 2018). Targeted sleep interventions may aid in the prevention and even reversal of cytotoxic levels of Aβ in pre-onset affected individuals (Ahmadian et al., 2018), particularly through the lens of MT and sleep quality.

Although the accumulative effects leading to neurodegeneration in PD involve different factors, sleep disorders and motor dysfunction still reign prevalent. Flattened melatonin rhythmicity, diurnal activity, and altered circadian gene expression are all symptoms of this neurodegenerative disorder (Sharma et al., 2020). However, one specific sleep disorder, REM behavioral disorder (RBD), is often considered a comorbidity and possible early indicator for the development of PD; 51% of PD patients exhibited RBD-related incidents during sleep, and also suffer from loss of non-REM SWS and excessive sleep fragmentation (Sixel-Döring et al., 2014). Maintaining the rhythmicity of sleep has been shown to be a legitimate concern for AD and PD patients along with a plethora of symptoms such as movement disorders, irritability, dementia, and cognitive decline.

## Music Therapy in The Context of Neurodegeneration

In order to understand the benefits of MT, we must explore how music-based interventions and rhythmic stimuli have been shown to improve the quality of life for affected individuals and functionally affect neural systems. Cost-effective and non-invasive, MT offers a pleasant and socially enhancing experience for patients undergoing therapy; pleasurability often accompanies MT, and the meaningful active engagement through MT may offer enhanced treatment adherence as compared to meaningless activities (Svansdottir and Snaedal, 2006). MT has also been shown to offer improvements in aggression, anxiety, and activity disturbances in AD patients regardless of disease severity (Svansdottir and Snaedal, 2006). Cognitive abilities are also improved in AD patients, despite varying levels of dementia severity (Gómez Gallego and Gómez García, 2017). The improvements of symptoms from MT exist as a multi-modal treatment outcome, where multiple symptomatic parameters are improved through music-based treatment rather than a specific ailment.

**Figure 1:**
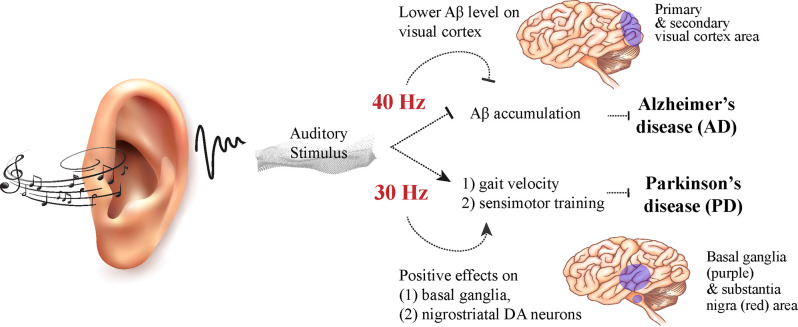
Illustration for the effects of MT-mediated auditory stimulus on neurodegenerative disorders. 40 Hz, as seen as deficient in gamma frequencies in AD patients, has been shown to markedly reduce Aβ accumulation in the visual cortex of mice. 30 Hz has shown improvement in gait velocity in PD patients. Manipulation of these frequencies in the context of MT or sound-based interventions may provide insight into other forms of treatment for AD and PD.

MT interventions and rhythmic auditory stimuli (RAS) carry an advantageous weight in the clinical setting, non-reliant on pharmacological methods which often may result in side effects. Clements-Cortes and Bartel (2018), aptly review advancements in the realm of MT for curtailing the progression of these neurodegenerative diseases and improving cognitive function through subjective and objective qualifications. Cognitive, emotional, and learned responses are particularly evident in treatment for individuals with AD and dementia; aggression, irritability, agitation, and wandering all are decreased after music interventions (Clements-Cortes and Bartel, 2018). Additionally, objective changes are observable in response to MT, evident in the improvement in gait velocity and stride length in response to RAS therapy for sensorimotor training in PD patients (Clements-Cortes and Bartel, 2018). Gamma rhythmicity can also be altered in many neurodegenerative disorders, where decreased gamma amplitudes at 40 Hz can be present in AD patients, and stimulation of interneurons with this exact frequency resulted in decreased levels of Aβ as well as increased microglial recruitment required to clear Aβ accumulation; striking reductions in Aβ levels were also observed in the visual cortex of pre-deposited Aβ mice (Iaccarino et al., 2016). Rhythmic Sensory Stimulus (RSS) in the form of pulsed vibrotactile whole body sound wave therapy at 30 Hz was shown to improve motor function in patients with PD specifically through step length, velocity, and decreases in tremor and rigidity (King et al., 2009). After exposing neural cells and nerve growth factor (NGF) to frequencies of 10–200 Hz, 40 Hz was found to be the most effective frequency resulting in observable neurite outgrowth three times the other frequencies in the range of 10–100 Hz (Koike et al., 2004). These specific frequencies, as seen in Figure 1, are crucial steps in demystifying the link through which auditory stimuli and ultimately even music have an effect on a molecular level.

Music is also implicated in affecting the structural integrity of brain areas involved with musical memory in AD patients (Jacobsen et al., 2015). Highlighted in the role of preserving musical memory are the caudal anterior cingulate and ventral pre-supplementary motor areas (Jacobsen et al., 2015). Cortical atrophy was found to be significantly less in these areas in individuals afflicted with AD along with minimally disrupted glucose metabolism (Jacobsen et al., 2015). Interestingly, the caudal anterior cingulate and ventral pre-supplementary motor areas exhibited Aβ levels characteristic of the rest of the cortically atrophied brain, indicating that total atrophy had not yet occurred, and these areas remain in early stages of degradation (Jacobsen et al., 2015). A neuroprotective role may exist in preserving complex memories in patients with AD, and music interventions utilizing familiar music have been shown to improve cognitive ability during the early stages of AD (Leggieri et al., 2018).

Establishing the results of MT as a treatment for AD is vital for understanding how these functional changes occur. However, the effects of rhythm-based therapies are more appropriate for PD due to the impairment of basal ganglia and destruction of nigrostriatal DA neurons, resulting in a lack of internally generated rhythmic motor cues (Thaut, 2015). Numerous mechanisms are suggested through which music aids PD patients, such as priming of the motor network to begin movement or RAS enhancing neuronal synchronization (Devlin et al., 2019). Cognitive rehabilitation and subjective enhancement are vital for improving the quality of life when faced with such debilitating circumstances. Six weeks of biweekly West African drumming circle therapy for PD patients significantly increased Parkinson Disease Questionnaire (PDQ-39) scores as well as self-reported improvements in movement (Pantelyat et al., 2016). RAS has been shown to improve Functional Gait Assessment scores and produce stronger EEG power during the gait cycle within sensorimotor rhythms, as well as enhanced functional connectivity between frontocentral parietal and temporal lobe connectivity (Calabrò et al., 2019). Due to the lack of internal cueing stimulus, the cerebellar network may often be recruited in compensation for the loss of basal network functionality in PD patients, resulting in altered connectivity, although the exact mechanism through which RAS compensates for this lack of internal cueing stimulus is unknown (Thaut, 2015; Braunlich et al., 2019). Auditory and motor inter-network connectivity is greatly enhanced in PD subjects unlike intra-network connectivity, which exhibits the contrary (Braunlich et al., 2019). Enhanced connectivity between auditory and visual, auditory and executive control, and motor cerebellar and executive control is also evident (Braunlich et al., 2019). RAS has the greatest effect on intra-network connectivity within the auditory areas, suggesting that compensation for lack of internal cueing stimulus may rely on this enhanced connectivity within auditory areas and between auditory, motor/cerebellar, and executive control areas (Braunlich et al., 2019).

Interestingly, much of this literature is concerned with the post-onset effects of music on ND and ameliorating mostly symptomatic ailments of the disease through subjective qualification. Longitudinal studies with homogeneous forms of MT are required to identify which concomitant forms of MT provide the best results along with current clinical treatment (García-Casares et al., 2018). Music has established a definitive role in affecting structural or connective alterations in neurodegenerative disorders as well as through functional and subjective improvement of symptoms in ND patients. However, in order to move forward with analyzing possible neurological mechanisms through which MT may alter the pathogenesis of ND, current literature, although limited, covering MT in the context of CR must be explored, namely through the analysis of music and its explicit effect on circadian processes.

Sleep exists as one of the most obvious manifestations of CR and severely impacts cognitive function; Loewy (2020) emphasizes the lack of a universal form of MT as sleep aids or proper consultation with music therapists to tailor the types of MT and musical excerpt selection to individual patients. Calming sleep-promoting elements must be emphasized rather than researcher bias, and previous musical engagement may change the receptiveness of an individual to the potential benefits of music sleep therapy (suggestibility; Loewy, 2020).

**Figure 2:**
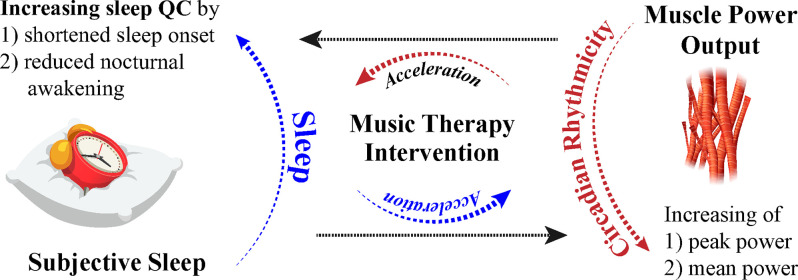
Illustration for the effects of MT intervention in the context of Sleep and CR.

Numerous parameters may serve beneficial in formulating diverse tailored music sleep therapy interventions with music therapists based on the history of sleep quality, history, and duration of music-based intervention (Loewy, 2020). Soft music, described as “sedative” by Lai and Good (2005), was used as a music intervention as a sleep aid for elderly individuals with difficulty sleeping. Sleep quality reported through the Pittsburgh Sleep Quality Index (PSQI) was higher than controls in the form of longer sleep duration and shorter sleep onset, and positive effects of the MT exhibited a compounding nature, improving sleep quality weekly (Lai and Good, 2005). Subjective improvements in sleep quality during a 90-min nap (shortened sleep onset and reduced nocturnal awakenings) are evident after MT; subjective sleep improves in individuals regardless of suggestibility, but objective improvements in sleep after MT in low suggestible individuals resulted in higher amounts of SWS and higher ratios of low to high sleep frequencies, indicating more restorative sleep (Cordi et al., 2019). Furthermore, studies suggest that music has the capacity to alter other physiological functions. Mean and peak power, as measured through the Wingate test, experience diurnal fluctuations at 7:00 h and 17:00 h and increase throughout the day (Chtourou et al., 2012). Individuals that participated in the warmups with music expressed higher mean and peak power in the morning compared to controls, implicating that music may have an effect on motor function and power output (Chtourou et al., 2012). Pianists specifically with late chronotypes, as evaluated through the Münich Chronotype Questionnaire, exhibited higher sensorimotor precision in the evening, further demonstrating the possible link between circadian fluctuations and highly complex sensorimotor tasks (Van Vugt et al., 2013). Interestingly, relaxing music has been found to improve symptoms of hypertonia during recovery periods after fatigue-inducing exercise, as measured by analyzing smoothness of movement and motor unit recruitment (Van Criekinge et al., 2021). The effects of MT on hypertonia in the form of rigidity in PD patients (Solopova et al., 2014) and other motor ailments have been shown to improve symptoms (Pacchetti et al., 2000), however, the specific circadian manifestations of these symptoms and timing of certain therapies in relation to the daily peak of symptoms have yet to be analyzed. The involvement between the timing of MT administration and the endogenous rhythms of physiological processes in the human body must be further explored in addition to the general effects of music on other physiological processes in neurodegenerative populations. However, music establishes itself as a viable potential non-pharmacological and cost-effective treatment to enhance circadian processes through subjective assessment and neurologically evident methods (Figure 2).

## Circadian-Based Musical Interventions as Treatment and Prevention for ND

A majority of the previously discussed literature analyses post-onset variabilities in responses to MT in populations afflicted with neurodegenerative disorders. Contrastingly, regarding music intervention as a circadian aid, mostly non-afflicted subjects have been utilized. The crossroads between mechanisms of MT to ameliorate sleep-related symptoms of ND patients remains largely unexplored, as well as the analysis of potential alterations in the pathogenesis of the ND in response to MT through a circadian or sleep-based lens. However, 4 weeks of 30–40-min morning sessions of music therapy for patients with AD resulted in marked increases in serum melatonin levels (Kumar et al., 1999), although attributed to a subjective improvement through the calmed mood of patients. With current literature emphasizing the interactions between CR and ND, this highly applicable finding helps solidify the proposed link between possible mechanisms through which MT may affect CR function in individuals with ND. As previously mentioned, diurnally fluctuating melatonin levels are often blunted in PD patients (Radogna et al., 2010), and MT may improve the blunted fluctuation.

Using music-based interventions in patients with AD may yield results by improving the objective parameters of SWS stages and reducing sleep onset. This critical function of Aβ clearance through increased GF may be enhanced due to the increase in SWS sleep from the music aid, particularly when the musical aids are utilized directly before sleep onset or at certain time markers during the day that promote relaxation and ease in ND patients. However, the knowledge of these theoretical time markers or optimal receptiveness and “suggestibility” to treatment in ND patients must be determined to optimize this method of utilizing MT in a circadian fashion. Furthermore, the effect of MT on Aβ level fluctuations and clearance must be analyzed in pre-onset individuals, healthy populations, or early-stage AD patients to possibly discover a mechanism through which music may affect AD pathogenesis. One is able to raise this question whether exposure to MT or other interventions prior to sleep-onset may improve SWS sleep quality in ND patients that may be experiencing circadian sleep disturbances, ultimately indirectly improving Aβ clearance, or if somehow MT itself may have a direct neurophysiological mechanism through which Aβ levels and proper clearance protocols are thoroughly regulated by previously unknown, unrelated, or even related neurodegenerative factors. The possibilities and nuances may even broaden when incorporating the concurrent use of 40 Hz frequencies along with musical aids in ND patients prior to sleep onset or during these theoretical daily time markers. The specified 40 Hz gamma frequencies that are often blunted in AD patients may also yield interesting results when utilized for patients with AD in respect to Aβ clearance and tau pathology as a concomitant form of intervention with sleep-based MT. The combination of MT and melatonin administration has also been yet to be observed in ND populations and unafflicted populations; results of this analysis may provide even further insight into how MT may enhance or “boost” current circadian-based treatments. Multiple combinations of factors when involving musical aid to treat AD and even RBD-afflicted PD patients through the lens of sleep are yet to be explored and may even herald the development of new possible treatment methods, both preventative and in response to disease pathology.

Contrastingly, many of the RAS/MT-involved studies rarely utilized a circadian parameter, such as time of day of the intervention, or analysis of circadian-related physiological indicators. Circadian symptomatic expression of PD patients is apparent through acrocyanosis, where patients that experienced this also exhibited diurnal fluctuations of other symptoms and increased falling, indicated by increased duration of PD diagnosis.

Music’s ability to affect the diurnal mean and peak power output may also shed insight on the potential timing of RAS-based intervention for the improvement of PD symptoms and slowing of DA neuronal degeneration. The flattened activity curve of PD may also indicate that RAS or upbeat forms of music therapy, such as group drumming, may be best performed in the morning to encourage movement and activation of movement-based neural structures affected by the disease. Many PD patients report that L-dopa treatment is most effective in the morning rather than evening (Li et al., 2017), suggesting that early RAS-induced priming of motor neural systems may complement L-dopa treatment to improve symptoms.

The implications of the use of MT as an intervention to ameliorate severe neurodegenerative disorders compound as subsequent literature is published. The lens through which music may aid afflicted individuals can be focused on the relationship between CR and ND, specifically diurnally fluctuating physiological functions such as sleep and movement. The potential of MT to affect the pathogenesis of AD and PD must be objectified and may provide insight into mechanisms that can be further utilized to develop standardized music-based circadian treatments for neuroprotection and neurodegenerative disorders.

## Key Concepts

**Music Therapy**—Non-pharmacological form of therapeutic intervention ranging from administration of musical excerpts or familiar songs to vibrotactile or rhythmic stimulus.

**Rhythmic Auditory Stimulus** employs the rhythmic and auditory characteristics of MT and has been found efficacious in the realm of PD (Clements-Cortes and Bartel, 2018).

**Circadian Rhythm**—Biologically evident rhythms that manifest in organisms from the molecular level. These rhythms are indicated by the free running period *tau* of approximately 24 h even in the absence of external cues (Sharma et al., 2020).

**Neurodegeneration**—Neurological process through which atrophy of brain areas or targeted neuronal cell death occurs. Pertinent examples of protein pathology dependent neurodegenerative diseases are Alzheimer’s and Parkinson’s Disease.

**Alzheimer’s Disease** can be characterized by AB accumulation and Tau protein aggregation resulting in neurodegeneration of larger areas of the brain.

**Parkinson’s Disease** displays targeted death of DA striatal neurons (Sharma et al., 2020).

## Author Contributions

AS, EM, and S-UK designed the review study. AS, EM, GK, and S-UK performed collecting references, data with experimental summary details, and wrote the article. All authors contributed to the article and approved the submitted version.

## Conflict of Interest

The authors declare that the research was conducted in the absence of any commercial or financial relationships that could be construed as a potential conflict of interest.

## Publisher’s Note

All claims expressed in this article are solely those of the authors and do not necessarily represent those of their affiliated organizations, or those of the publisher, the editors and the reviewers. Any product that may be evaluated in this article, or claim that may be made by its manufacturer, is not guaranteed or endorsed by the publisher.
